# Supplementary material for: Poly(β-cyclodextrin)-Activated Carbon Gel Composites for Removal of Pesticides from Water
Source: Molecules. 2021 Mar 6;26(5):1426. doi: 10.3390/molecules26051426 (PMC7962014; doi:10.3390/molecules26051426)
Supplement: Supplementary file 1 [file molecules-26-01426-s001.pdf]

molecules

MDPI

Supplementary Material

Poly(β-cyclodextrin)-activated carbon gel composites for removal of pesticides from water

Gianluca Utzeri[1], Luis Verissimo[1], Dina Murtinho[1], Alberto A.C.C. Pais[1], F. Xavier Perrin[2], Fabio Ziarelli[3], Tanta-Verona Iordache[4], Andrei Sarbu[4] and Artur J.M. Valente[1,*]

[1] University of Coimbra, Coimbra Chemistry Centre, Department of Chemistry, 3004–535 Coimbra, Portugal; uc2015276036@student.uc.pt; luisve@gmail.com; dmurtinho@ci.uc.pt; pais@ci.uc.pt; avalente@ci.uc.pt

[2] Université de Toulon, Laboratoire MAPIEM, 83041 Toulon CEDEX 9, France; perrin@univ-tln.fr

[3] Aix Marseille University, CNRS, Centrale Marseille, FSCM, Marseille, France; fabio.ziarelli@univ-amu.fr

[4] National Institute for Research & Development for Chemistry and Petrochemistry- ICECHIM, Splaiul Independenței 202, București, 060021, Romania; iordachev.icechim@gmail.com; andr.sarbu@gmail.com

Table 1. One-tailored Grubb's test parameters.

Imidacloprid					
Theorical Concentration (mg L^{-1})	Average Concentration (mg L^{-1})	Standard Deviation	G calculated		G table*
			Inferior	Superior	
0.5	0.502	0.0003	0.98	1.02	2.21
1	1.004	0.0004	0.80	1.12	
2.5	2.51	0.0011	0.97	1.03	
5	5.02	0.0003	0.94	1.05	
7.5	7.53	0.0002	0.86	1.10	
10	10.04	0.002	0.85	1.10	
15	15.06	0.0004	1.11	0.84	
20	20.08	0.002	0.87	1.09	
**Cymoxanil**					
Theorical concentration (mg L^{-1})	Average concentration (mg L^{-1})	Standard deviation	G calculated		G table*
			Inferior	superior	
0.1	0.0989	0.0005	0.71	1.14	2.29
0.5	0.4945	0.0001	1.15	0.62	
1	0.9890	0.0004	1.07	0.91	
2.5	2.4725	0.0003	1.01	0.98	
5	4.9450	0.0009	0.86	1.10	
7.5	7.4175	0.0004	1.05	0.94	
10	9.8900	0.0007	1.05	0.93	
15	14.8350	0.0004	1.05	0.94	
20	19.780	0.001	1.05	0.94	
30	29.6700	0.0003	0.58	1.15	

*significance level (α) = 0.05.

Table 2. Two-tailored F-test parameters for equality variance.

Imidacloprid		Cymoxanil	
**F calculated**	$F_{(\alpha-2, N1-1, N2-1)}$ [*,a]	**F calculated**	$F_{(\alpha-2, N1-1, N2-1)}$ [*,b]
0.08	8.89	0.32	6.54

[*] significance level (α) = 0.01

[a] degrees of freedom (N) = 8

[b] degrees of freedom (N) =10

Table 3. Analytical parameters of imidacloprid and cymoxanil.

Pesticide	LOD (mg L^{-1})	LOQ (mg L^{-1})	ε (10^{-2} M^{-1} cm^{-1})	R^2
**imidacloprid**	0.05	20.08	8.38 (±0.04)	0.9997
**cymoxanil**	0.07	29.67	4.08 (±0.05)	0.9983

Table 4. Fitting parameters for eqs. 4 and 5 to the sorption isotherm data for imidacloprid in the presence of urea and NaCl.

		**urea, 1.0 g L^{-1}**			
**Model**		**PCD**	**$PCD/AC_{5\%}$**	**$PCD/AC_{10\%}$**	**AC**
Freundlich	K_F (mg g^{-1} $mg^{-1/n}$ $L^{1/n}$)	0.08 (±0.01)	0.63 (±0.05)	1.43 (±0.22)	18.26 (±2.57)
	1/n	0.94 (±0.03)	0.64 (± 0.016)	0.52 (± 0.03)	0.82 (± 0.13)
	R^2	0.996	0.9977	0.985	0.9245
	AIC	-12.10	-15.42	2.57	31.40
Sips	q_m (mg g^{-1})	44.80 (±15.74)	36.70 (±9.60)	32.60 (±8.84)	55.00 (±2.40)
	K_S (10^{-2} L mg^{-1})	0.22 (±0.11)	0.53 (±0.30)	1.03 (±0.72)	72.75 (±3.64)
	$1/n_S$	1.21 (±0.13)	1.97 (±0.15)	0.82 (±0.15)	2.51 (±0.24)
	R^2	0.997	0.9913	0.9062	0.9957
	AIC	-13.82	-0.33	3.45	10.76
		NaCl, 1.0 g L^{-1}			
Model		PCD	$PCD/AC_{5\%}$	$PCD/AC_{10\%}$	AC
Freundlich	K_F (mg g^{-1} $mg^{-1/n}$ $L^{1/n}$)	0.063 (±0.047)	0.32 (±0.08)	1.35 (± 0.27)	7.86 (±1.82)
	1/n	0.98 (± 0.14)	0.68 (±0.04)	0.52 (±0.04)	1.31 (±0.21)
	R^2	0.9441	0.9834	0.9811	0.9225
	AIC	8.15	-1.33	7.70	21.02
Sips	q_m (mg g^{-1})	15.43 (±0.64)	30.90 (±10.11)	34.80 (±7.60)	40.42 (± 2.50)
	K_S (10^{-2} L mg^{-1})	0.90 (±0.040)	0.35 (±0.22)	0.87 (±0.50)	62.03 (±2.61)
	$1/n_S$	2.73 (±0.23)	1.02 (±0.17)	0.85 (±0.14)	6.23 (±1.76)
	R^2	0.9976	0.9932	0.9927	0.981
	AIC	-14.19	-6.13	0.85	16.01

Table 5. Fitting parameters for eqs. 4 and 5 to the sorption isotherm data for cymoxanil in presence of urea and NaCl.

**urea, 1.0 g L⁻¹**					
	**Model**	**PCD**	**PCD/AC$_{5\%}$**	**PCD/AC$_{10\%}$**	**AC**
Freundlich	K_F (mg g^{-1} mg$^{-1/n}$ L$^{1/n}$)	0.03 (± 0.01)	0.53 (± 0.15)	2.26 (± 0.30)	5.90 (±1.70)
	1/n	0.87 (± 0.09)	0.51 (± 0.05)	0.32 (±0.02)	0.93 (±0.16)
	R^2	0.9687	0.959	0.9765	0.8990
	AIC	-9.83	-2.19	-3.93	25.60
Sips	q_m (mg g^{-1})	9.54 (±6.31)	13.25 (±2.70)	20.61 (±4.18)	44.01 (±3.02)
	K_S (10^{-2} L mg^{-1})	0.27 (±0.27)	0.90 (±0.44)	1.09 (±0.77)	28.28 (±1.70)
	$1/n_S$	1.36 (±0.52)	0.98 (±0.200)	0.62 (±0.12)	3.34 (±0.56)
	R^2	0.9725	0.9835	0.9951	0.9852
	AIC	-10.23	-8.94	-11.34	15.90
NaCl, 1.0 g L⁻¹					
	Model	PCD	PCD/AC$_{5\%}$	PCD/AC$_{10\%}$	AC
Freundlich	K_F (mg g^{-1} mg$^{-1/n}$ L$^{1/n}$)	0.08 (± 0.03)	0.15 (± 0.02)	0.72 (±0.10)	4.80 (±0.70)
	1/n	0.86 (± 0.07)	0.80 (±0.03)	0.60 (±0.03)	1.58 (±0.10)
	R^2	0.973	0.9943	0.9932	0.9902
	AIC	0.05	-12.70	-4.12	14.53
Sips	q_m (mg g^{-1})	18.58 (±2.80)	28.84 (±5.07)	35.73 (±8.04)	76.06 (±32.25)
	K_S (10^{-2} L mg^{-1})	0.47 (±0.11)	0.32 (±0.97)	0.44 (±0.22)	27.95 (±10.76)
	$1/n_S$	1.50 (±0.20)	1.15 (±0.10)	0.89 (±0.11)	2.40 (±0.64)
	R^2	0.9932	0.9972	0.9955	0.983
	AIC	-11.00	-19.90	-6.60	21.65

Table 6. Characterization values for three sorption/desorption cycles.

	PCD			PCD/AC$_{5\%}$			PCD/AC$_{10\%}$		
IMD	q_e **(mg g^{-1})**	***RE%***	***DC%***	q_e **(mg g^{-1})**	***RE%***	***DC%***	q_e **(mg g^{-1})**	***RE%***	***DC%***
1° cycle	15.0 (±0.3)	33.3 (±0.3)	64 (±2)	20.8 (±0.3)	43.2 (±0.8)	45 (±1)	24.0 (±0.3)	49.0 (±0.4)	31.2 (±0.4)
2° cycle	17.0 (±0.5)	36 (±1)	71 (±2)	23.6 (±0.5)	50 (±1)	51 (±1)	32.0 (±0.5)	66 (±1)	37.0 (±0.6)
3° cycle	18.3 (±0.6)	38 (±1)	70 (±2)	24.7 (±0.6)	50.3 (±1.3)	51 (±1)	34.0 (±0.6)	69 (±1)	36.4 (±0.7)
CYM	q_e **(mg g^{-1})**	***RE%***	***DC%***	q_e **(mg g^{-1})**	***RE%***	***DC%***	q_e **(mg g^{-1})**	***RE%***	***DC%***
1° cycle	6.3 (±0.9)	11.8 (±0.2)	54 (±8)	13.9 (±0.9)	26 (±2)	42 (±3)	18.8 (±0.8)	35 (±2)	34 (±2)
2° cycle	9.8 (±1.3)	18 (±2)	36 (±5)	18 (±1)	33 (±2)	32 (±2)	24 (±1)	44 (±2)	30 (±2)
3° cycle	10.5 (±1.6)	21 (±3)	35 (±5)	18.9 (±1.5)	37 (±3)	32 (±3)	23 (±2)	46 (±3)	31 (±2)

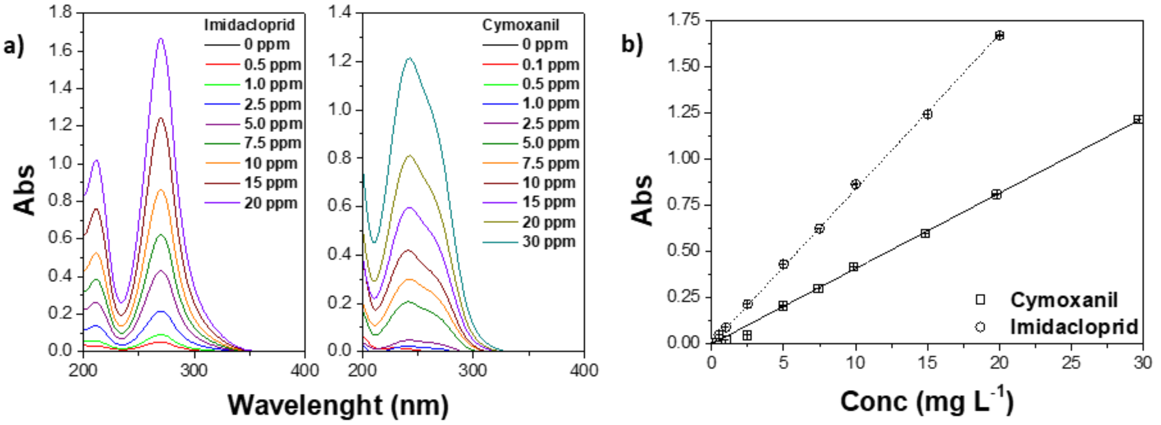

Figure 1. Representation of IMD and CYM (a) UV-Vis spectra, and (b) curve of calibration.

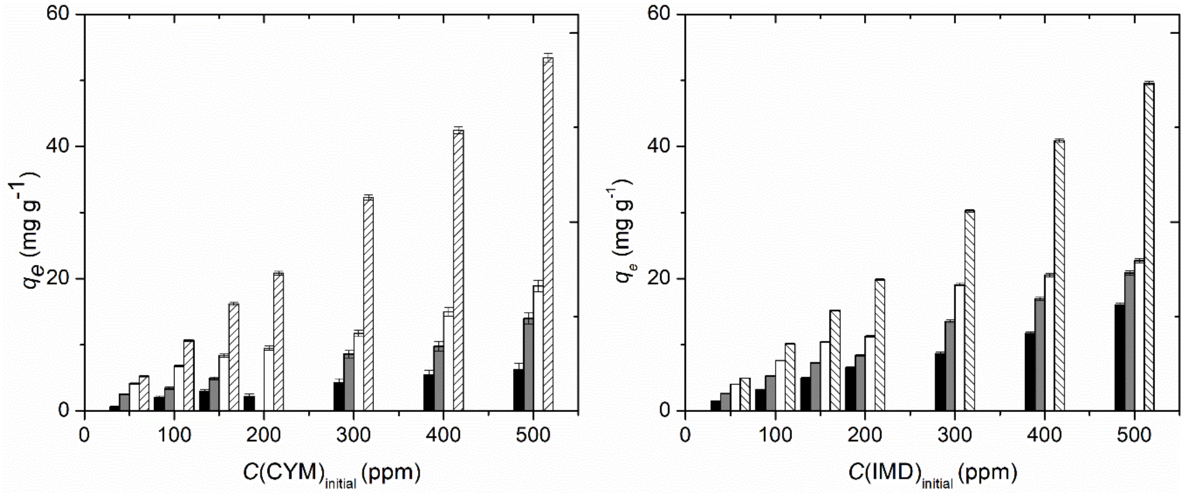

Figure 2. Maximum sorbed amount (q_e) of CYM (left) and IMD (right) by different adsorbents: PCD (black columns), PCD/AC$_{5\%}$ (grey), PCD/AC$_{10\%}$ (white) and AC (lines).

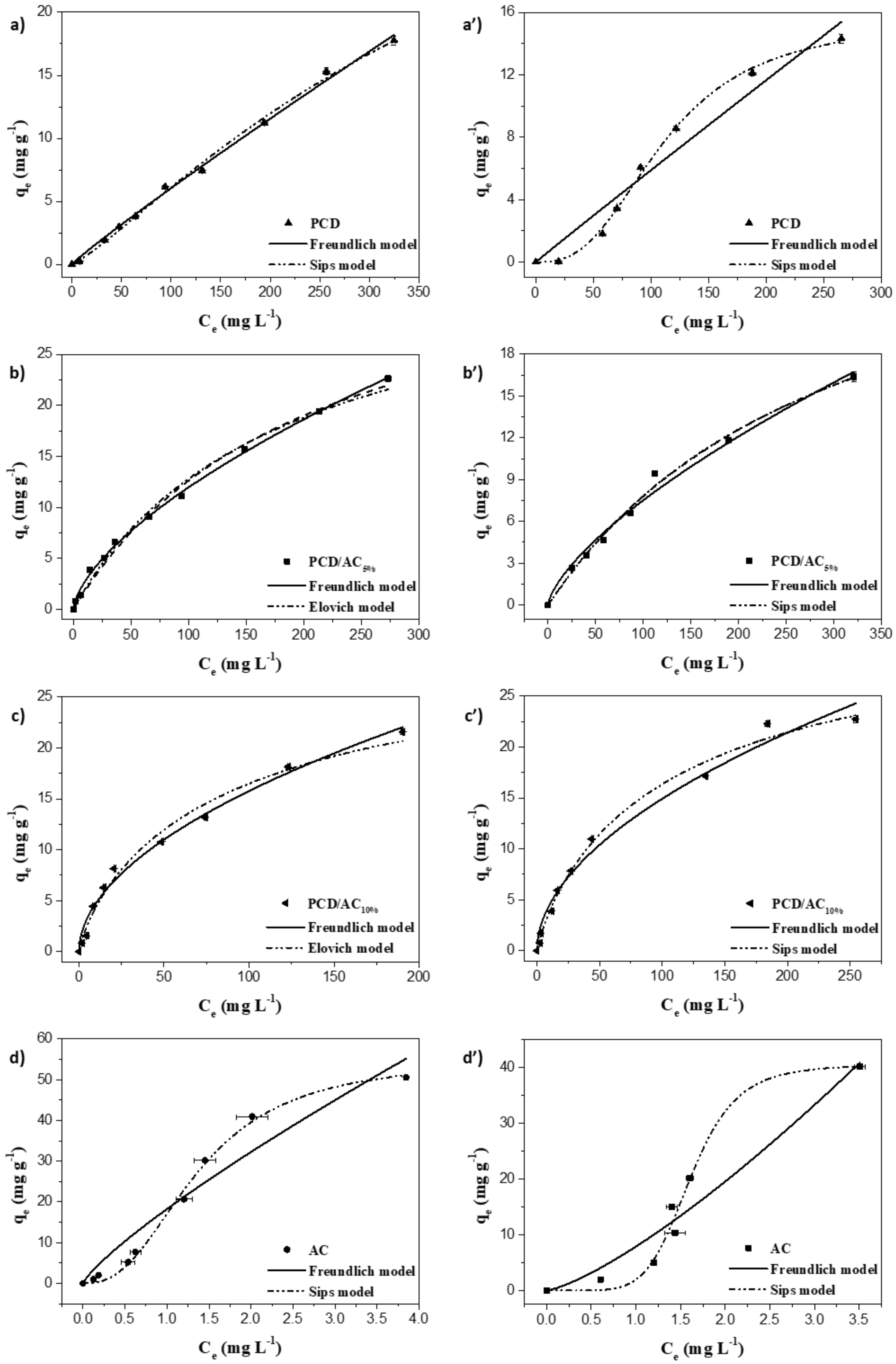

Figure 3. Adsorption isotherm of IMD (0-500) ppm onto PCD (a), PCD/AC$_{5\%}$ (b), PCD/AC$_{10\%}$ (c) and AC (d), in 1.0 g L^{-1} urea solution; and onto PCD (a′), PCD/AC$_{5\%}$ (b′), PCD/AC$_{10\%}$ (c′) and AC (d′), in 1.0 g L^{-1} NaCl solution, at 25 °C.

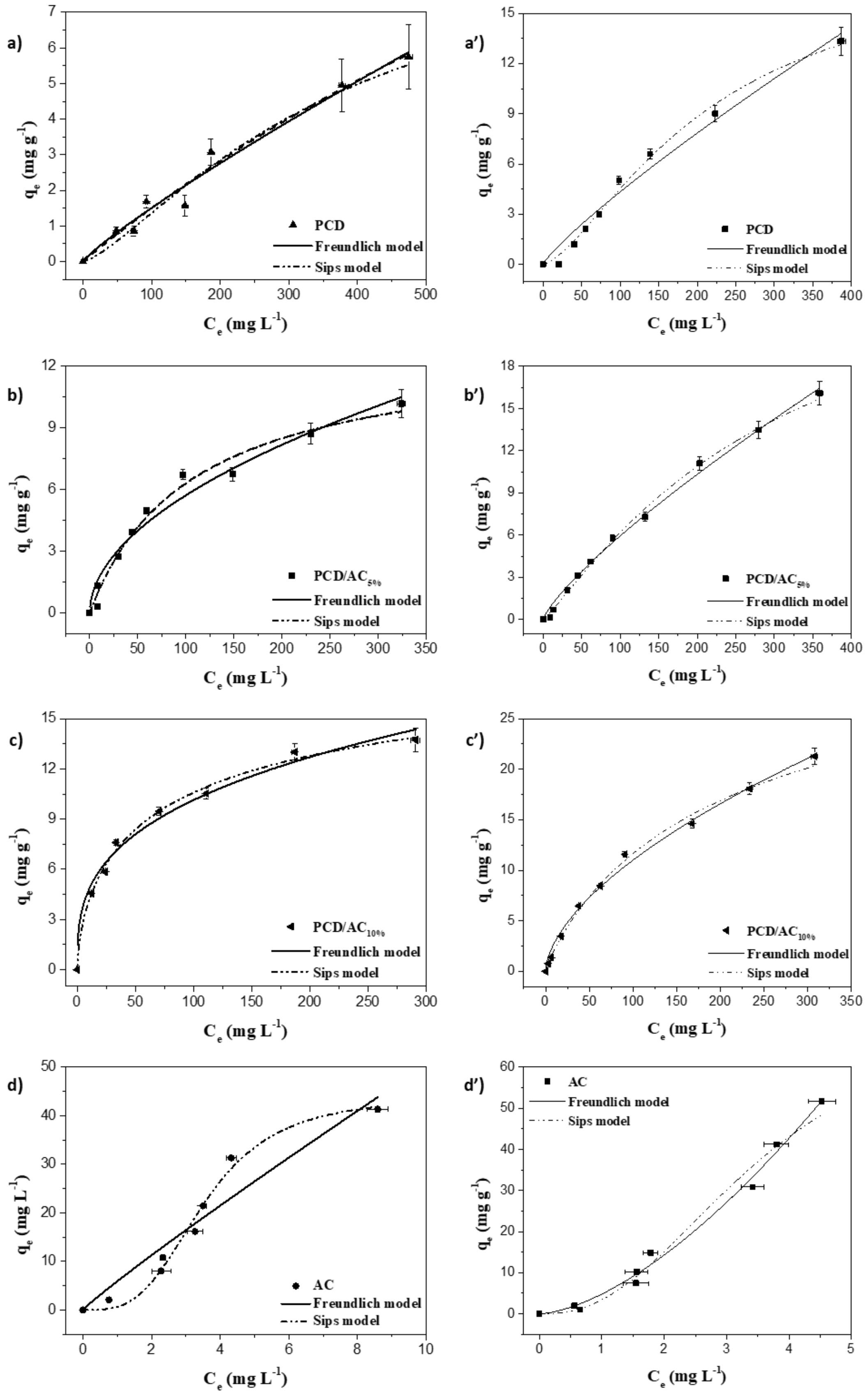

Fig. S4. Adsorption isotherm of CYM (0-500) ppm onto PCD (a), PCD/AC$_{5\%}$ (b), PCD/AC$_{10\%}$ (c) and AC (d), in 1.0 g L^{-1} urea solution; and onto PCD (a′), PCD/AC$_{5\%}$ (b′), PCD/AC$_{10\%}$ (c′) and AC (d′), in 1.0 g L^{-1} NaCl solution, at 25 °C.
